# Evaluation of Quality of Life After Use the Virtual Reality in Patients with Neurodegenerative Disease

**DOI:** 10.1055/s-0044-1785681

**Published:** 2024-04-30

**Authors:** Jéssica Spricigo Malisky, Bianca Lopes Cavalcante-Leão, Maria Izabel Severiano, Geslaine Janaina Bueno dos Santos, Hélio Augusto Guizoni Teive, Maria Renata José, Cristiano Miranda de Araújo, Bianca Simone Zeigelboim

**Affiliations:** 1 Department of Otoneurology, Postgraduate Program in Communication Disorders, Universidade Tuiuti do Paraná (UTP), Curitiba, PR, Brazil; 2 Department of Physical Therapy, Instituto Federal do Paraná, Curitiba, PR, Brazil; 3 Department of Neurology, Movement Disorders Unit, Hospital das Clínicas, Universidade Federal do Paraná, Curitiba, PR, Brazil

**Keywords:** Ataxia, quality of life, rehabilitation, neurodegenerative diseases, postural balance

## Abstract

**Introduction** Spinocerebellar ataxias (SCAs) are a heterogeneous group of neurodegenerative diseases.

**Objective** To evaluate the living standard of patients with SCA, by applying the Vestibular Disorders Activities of Daily Living Scale (VADL) and Activitiesspecific Balance Confidence Scale (ABC) questionnaires.

**Methods** An uncontrolled clinical trial study was conducted with 28 patients who underwent anamnesis, ENT evaluation, and vestibular assessment and the application of questionnaires VADL and ABC before and after rehabilitation with virtual reality.

**Results** The vestibular exam was altered in 64.3% of the cases. The result between the correlation of the VADL and ABC questionnaires showed significant results in all cases (p < 0.005). The correlation between the ages and disease length with the VADL and ABC questionnaires was significant in the T3 assessment (p = 0.015). The correlation between the disease length and the VADL questionnaire was significant in all cases (p < 0.005). The comparison of the vestibular rehabilitation result (T1 to T2) showed a significant difference for all the applied games, except for the ski slalom. The comparison of the vestibular rehabilitation result (T1 to T3) showed significant difference for all the applied games (p < 0.005) (1st assessment before the start of rehabilitation designated T1, after 10 rehabilitation sessions, considered T2 and, at the end of 20 rehabilitation sessions, called T3).

**Conclusion** We can point out a direct improvement in the living standard, reflected by the reduction of falls, better balance, and march, contributing to a higher self-confidence in patients in daily activities.

## Introduction

Spinocerebellar ataxias (SCAs) are a heterogeneous group of neurodegenerative diseases, especially progressive cerebellar ataxia. They present classical symptoms, such as ataxia, muscle hypotonia, nystagmus, dysarthria, and movement tremor. 1 2 3

The risk of falls is high and frequent in people with SCAs. The body balance depends on the vestibular system integrity: labyrinth, vestibulocochlear nerve, central nervous system (CNS) nuclei pathways and interrelationships, somatosensory system (sensory receptors in tendons, muscles, and joints), and vision. 2 4

Dizziness and/or imbalance may impair the individuals' performance of activities in daily life (ADLs), such as those that demand quick head movements and tasks that demand trunk and head flexion. 5 The extent to which dizziness impairs individuals' ADLs can be assessed by validated questionnaires. Currently, there are questionnaires that assess perceptions of dizziness and body balance to quantify the impacts of such symptoms on the patient's daily life. Among the existing instruments, the *Vestibular Activities of Daily Living Scale* (VADL), adapted to the Brazilian population by Aratani et al., 6 assesses a scale of the patients' self-perception on their performance and independence to conduct daily activities. Another instrument, the *Activities-Specific Balance Confidence Scale* (ABC), adapted to the Brazilian population by Marques et al., 7 verifies by a scale the individuals' self-confidence to conduct ADLs.

The CNS promptly organizes and processes visual, vestibular, and proprioceptive sensory information in specific areas in the brainstem and cerebellum, which control head, neck, cervical spine, legs, arms, eyes, and all body muscles. We stress the importance of the vestibular system in SCAs. 8

Vestibular rehabilitation (VR) is considered a therapeutic resource due to its proposal based on the central mechanisms of neuroplasticity. 4 9 10 The CNS processes that information and generates responses by means of reflexes. The vestibule-ocular reflex (VOR), allows visual stabilization during the head movements, and the vestibulospinal reflex (VSR), generates a body compensation movement to keep head and body stability, thus preventing falls. 4

Exercises aim to improve the visual-vestibular interaction during head motion and expand static and dynamic postural stability – because they produce conflicting sensory information –, enhance the capability to perform daily activities, and provide greater physical independence to patients. 10 11

Therefore, this study aims to evaluate the benefit of VR with virtual reality through the application of the VADL and ABC questionnaires in patients with SCA.

## Methods

The study fulfilled the Helsinki Declaration for Ethical use of human material. This study was approved by the Ethics Board (Plataforma Brasil) under no. 832.502/2014, CAAE 37083714.0.0000.0103. The anonymity of participants was guaranteed, and informed consent was obtained from all participants.

A descriptive, retrospective cross-sectional study was carried out. Twenty-eight patients (eight females and 20 males) (three SCA type 2, five SCA type 3, five SCA type 10, and eight SCA recessive type). Seven patients were under genetic investigation to diagnose the type of SCA that comprised the undetermined SCA type.

The SCA diagnosis was done by PCR (Polymerase Chain Reaction). 12 13 The PCR reaction is because oligonucleotides (primers) hybridize specifically with a DNA template strand, enabling the production of multiple copies of specific DNA sequences. 14

To measure the severity of ataxia, the Scale for the Assessment and Rating of Ataxia (SARA) proposed by Schmitz - Hübsch et al., 15 further translated and validated in Brazil by Braga-Neto et al. 16 was used.

Patients' age ranged from 15 to 70 years (mean age 41.6 ± 16.9 years). Disease onset ranged from two to 40 years (mean of 13.3 ± 12.4 years).

Patients included in the study: those with no middle-ear disorders, absence of assistive gait devices, and no former rehabilitation therapy. Patients excluded from the study: those unable to respond and understand simple verbal commands and severe visual impairment or other abnormalities that hindered the performance of the proposed procedures.

### Limitation of the Study

The study began with 35 patients. However, seven patients did not conclude the study (three died, two missed the therapy sessions, and two did not meet the inclusion criteria). We point to the difficulty in adhering this population to exercise since it is a highly impacting disease not only physically, but also psychologically.

### Measurements

All patients underwent an otorhinolaryngological exam, anamnesis, and vestibular assessment with digital vector electronystagmography (VENG) which has its own software VecWin2 (Neurograff Eletromedicina LTDA, São Paulo/SP, Brazil), a rotating chair model COD 14200, rotation 0.01 to 0.5Hz, (Cadeiras Ferrante Ltda, São Paulo, SP, Brazil), a visual stimulator model EV VEC (Neurograff Eletromedicina LTDA, São Paulo, SP, Brazil), and an air caloric stimulator model NGR 05 (Neurograff Eletromedicina LTDA, São Paulo, SP, Brazil). The flow rate used was 5 and 13 L/min. The VADL and ABC questionnaires were applied before rehabilitation: 1 ^st^ assessment (T1) was after ten rehabilitation sessions, the 2 ^nd^ assessment (T2) was at the end of 20 rehabilitation sessions, and the 3 ^rd^ assessment (T3) aimed to observe prospective changes after intervention.

### Vestibular Disorders Activities of Daily Living Scale (VADL)

This questionnaire assesses the dependence and the impacts of body imbalance on the performance of daily activities. The questionnaire includes 28 ADLs divided into three subscales: functional (12 activities), ambulation (nine activities), and instrumental (seven activities). Using a qualitative scale, the patients scored 0 to 10 points according to their performance and independence in performing each described activity. The score is calculated by median values: the higher the score, the higher the dependence and inability of the patient. For “not applicable” (N/A) answers, that is, if the patient does not perform the activity or does not want to reply, the question is given a score of zero.

### Activities-specific Balance Confidence Scale (ABC)

This questionnaire assesses the level of confidence of the individual in the ability to maintain balance while performing specific daily activities. The questionnaire comprises 16 questions on how confident subjects are to conduct a certain activity (not confident 0%, and completely confident to conduct the activity without losing balance 100%). Therefore, the higher the percentage, the greater the self-confidence.

### Vestibular Rehabilitation with Virtual Reality

This is a therapeutic method used in body balance rehabilitation. A Nintendo ^®^ Wii was used: Wii-Remote and Wii Balance Board (Nintendo Co, Ltd., Kyoto, Japan) were used. This platform measures the applied strength and senses balance changes by pressure sensors. The sensors are responsible for the interface between the machine and the player. Initially, the patients got used to the game and were instructed on the necessary movements to play it.

Four balance games were played (Soccer Heading, Table Tilt, Tightrope, and Ski Slalom). Strategies aimed at training gaze stability, head movement, static and dynamic balance, motor coordination, rotation movement of the pelvis, and weight transfer aiming to verify changes in balance and postural stability. All patients underwent 20 sessions of VR of 50 minutes each twice a week. Then, they answered the same assessment questionnaires before and after the end of rehabilitation sessions.

### Statistical Analysis

Comparisons between groups and times were made using Friedman's non-parametric test/two-way analysis of variance by ranks (Friedman's ANOVA). To compare two nominal variables in VADL and ABC questionnaires, the Wilcoxon signed-rank test was used considering Bonferroni correction.

Spearman's correlation was used to analyze the correlation between disease time and the VADL and ABC questionnaires. The correlation strength between these variables was considered very strong, strong, moderate, low, and irrelevant, when the values of the correlation coefficient (positive or negative) were between 0.9 - 1, 0.7 - 0.89, 0.5 - 0.69, 0.3 - 0.49, and 0.0–0.29, respectively. 17

A comparison of results in games and their interaction with group X time was made by Student paired t-test.

All statistical analyses were conducted using the software SPSS v. 16.0 (IBM SPSS, Armonk, NY) and Statistical 6.0 with a significance level of 5%.

## Result

The most reported otoneurologic complaints in the anamnesis were imbalance (85.7%), falls (28.5%), dizziness (17.8%), diplopia (10.7%), and tremor (7.1%).

The vestibular exam pointed to disorders in 18 cases (64.4%), with ten cases (35.8%) of peripheral vestibular disorders and eight cases (28.6%) of central vestibular disorders. Testing was normal in ten cases (35.6%).

Friedman's ANOVA showed that the comparison group x time of the VADL questionnaire for the functional (p = 0.236), ambulation (p = 0.936), and instrumental (p = 0.973) domains had no significant differences between the scores of the three domains (p > 0.05). Comparison between the total scores of the VADL questionnaire also showed no significant differences (p = 0.531). The comparison of two nominal variables by the Wilcoxon signed-rank test did not show statistically significant differences (p > 0.005) ( Table 1 ).

**Table 1:** Comparisons of the results of the VADL and ABC questionnaire according to the evaluation time (n = 28)

Evaluation time: Mediana (IQR)			Valor de p*		
1st assessment (F)	2st assessment (F)	3st assessment (F)	T1 x T2	T1 x T3	T2 x T3
1.00 (1.00–3.75)	1.00 (1.00–2.75)	1.00 (1.00–1.00)	0.125	0.102	0.833
1st assessment (A)	2st assessment (A)	3st assessment (A)	T1 x T2	T1 x T3	T2 x T3
3.00 (1.00–7.75)	2.00 (1.00–7.75)	2.00 (1.00–8.75)	0.929	1.00	0.944
1st assessment (I)	2st assessment (I)	3st assessment (I)	T1 x T2	T1 x T3	T2 x T3
6.00 (1.00–10.00)	4.00 (1.00–10.00)	1.00 (1.00–10.00)	0.823	0.516	0.778
1st assessment (T)	2st assessment (T)	3st assessment (T)	T1 x T2	T1 x T3	T2 x T3
4.00 (1.00–8.00)	1.75 (1.00–8.37)	1.00 (1.00–8.37)	0.753	0.789	0.332
1st assessment (ABC)	2st assessment (ABC)	3st assessment (ABC)	T1 x T2	T1 x T3	T2 x T3
43.75 (24.84–79.06)	41.88 (15.93–86.56)	56.25 (17.96–80.46)	0.767	0.121	0.123

A: Ambulation; ABC: Activities-Specific Balance Confidence; F: Functional; I: Instrumental; IQR: Interquartile range (IQR, 25th to 75th percentile); T: Total; VADL: Vestibular Disorders Activities of Daily Living Scale.

* Wilcoxon signed-rank test.

Friedman's ANOVA evidenced no significant differences between the scores in the three assessments (T1, T2, and T3) (p = 0.119) compared to the interaction group x time in the ABC questionnaire. The comparison of two nominal variables by the Wilcoxon test also showed no statistical differences between times (p > 0.005) ( Table 1 ).

The application of Spearman correlation showed significant results (p < 0.005) between the VADL and ABC questionnaires in all cases ( Table 2 ). As the correlation coefficient was always negative, there was an inverse correlation between questionnaires, that is, lower scores in the VADL questionnaire correspond to higher scores in the ABC questionnaire. Correlation strengths ranged from strong to moderate between the different variables and are available in Table 2 . Therefore, the best results in the VADL questionnaire correspond to the best results in the ABC questionnaire. We can also notice that the greatest correlations between questionnaires occurred in the T3 assessment. Concerning dimensions, the greatest correlations were for the functional dimension.

**Table 2:** Correlation between VADL and ABC questionnaires (n = 28)

RESULTS	_rs	p*	CS
VADL functional and ABC 1st assessment (T1)	-0.762	*0.000	Strong
VADL ambulation and ABC 1st assessment (T1)	-0.662	*0.000	Moderate
VADL instrumental and ABC 1st assessment(T1)	-0.679	*0.000	Moderate
VADL general and ABC 1st assessment (T1)	-0.733	*0.000	Strong
VADL functional and ABC 2st assessment (T2)	-0.707	*0.000	Strong
VADL ambulation and ABC 2st assessment (T2)	-0.715	*0.000	Strong
VADL Instrumental and ABC 2st assessment (T2)	-0.549	*0.002	Moderate
VADL general and ABC 2st assessment (T2)	-0.718	*0.000	Strong
VADL functional and ABC 3st assessment (T3)	-0.758	*0.000	Strong
VADL ambulation and ABC 3st assessment (T3)	-0.733	*0.000	Strong
VADL instrumental and ABC 3st assessment(T3)	-0.740	*0.000	Strong
VADL general e ABC 3st assessment (T3)	-0.778	*0.000	Strong

ABC: Activities-Specific Balance Confidence Scale; CS: Correlation strength; VADL: Vestibular Disorders Activities of Daily Living Scale.

Significant p values are in bold *_rs test

The application of the Spearman correlation showed no significant correlations between ages and the VADL and ABC questionnaires, except for the instrumental dimension of the VADL questionnaire in the T3 assessment (p = 0.015) ( Table 3 ). As for disease time, the correlation was significant in all cases. As the coefficient correlation was always positive for the VADL questionnaire, there was a direct correlation to disease time, that is, the questionnaire results worsened as the disease time increased. Regarding the ABC questionnaire, all correlations were negative and there was an inverse correlation to disease time, that is, the worst questionnaire results in the ABC questionnaire are associated with a longer disease time. Correlation strengths for each variable are available in Table 3 .

**Table 3:** Correlation between age and length of the disease to the VADL and ABC questionnaires (n = 28)

RESULTS	AGE			LENGTH OF THE DISEASE		
	_rs	p*	CS	_rs	p*	CS
VADL functional 1st assessment (T1)	0.292	0.125	Irrelevant	0.529	*0.003	Moderate
VADL functional 2st assessment (T2)	0.244	0.201	Irrelevant	0.506	*0.006	Moderate
VADL functional 3st assessment (T3)	0.247	0.205	Irrelevant	0.400	*0.034	Low
VADL ambulation 1st assessment (T1)	0.157	0.422	Irrelevant	0.519	*0.004	Moderate
VADL ambulation 2st assessment (T2)	0.235	0.226	Irrelevant	0.502	*0.006	Moderate
VADL ambulation 3st assessment (T3)	0.304	0.115	Low	0.582	*0.001	Moderate
VADL instrumental 1st assessment (T1)	0.323	0.093	Low	0.535	*0.003	Moderate
VADL instrumental 2st assessment (T2)	0.343	0.073	Low	0.397	*0.036	Low
VADL instrumental 3st assessment (T3)	0.470	*0.011	Low	0.381	*0.045	Low
VADL general 1st assessment (T1)	0.255	0.189	Irrelevant	0.509	*0.005	Moderate
VADL general 2st assessment (T2)	0.259	0.182	Irrelevant	0.486	*0.008	Low
VADL general 3st assessment (T3)	0.365	0.055	Low	0.523	*0.004	Moderate
ABC 1st assessment (T1)	-0.218	0.263	Irrelevant	-0.503	*0.006	Moderate
ABC 2st assessment (T2)	-0.182	0.351	Irrelevant	-0.493	*0.007	Low
ABC 3st assessment (T3)	-0.222	0.254	Irrelevant	-0.454	*0.015	Low

ABC: Activities-Specific Balance Confidence; CS: Correlation strength; VADL: Vestibular Disorders Activities of Daily Living Scale.

Significant p values are in bold * _rs test

The comparison of the games played during the VR with virtual reality in T1 and T2 assessments by Student t-test showed significant differences for the games Soccer Heading (p = 0.006), Tightrope (p≤0.000), and Table Tilt (p = 0.000), except for the Ski Slalom (p = 0.100) ( Table 4 ).

**Table 4:** Virtual reality game performance (n = 28)

Game	Number of sessions (Mean ± Standard Deviation)			p*	
Soccer Heading	1st assessment (T1)	10th assessment (T2)	20th assessment (T3)	T1 x T2	T1 x T3
	27.3 ± 21.8	48.0 ± 41.6	58.7 ± 64.0	*0.006	*0.015
Tightrope	1st assessment (T1)	10th assessment (T2)	20th assessment (T3)	T1 x T2	T1 x T3
	12.5 ± 8.5	19.0 ± 12.1	24.0 ± 13.3	*0.000	*0.000
Table Tilt	1st assessment (T1)	10th assessment (T2)	20th assessment (T3)	T1 x T2	T1 x T3
	29.3 ± 13,5	40.5 ± 18.2	54.7 ± 29.6	*0.000	*0.000
Ski Slalom	1st assessment (T1)	10th assessment (T2)	20th assessment (T3)	T1 x T2	T1 x T3
	85.4 ± 18.5	76.5 ± 26.6	70.0 ± 24.3	0.100	*0.017

Significant p values are in bold *Paired Student's t-test

The comparison between T1 and T3 assessments regarding the games played during the VR with virtual reality by Student t-test showed significant differences in all applied games. There was a major improvement in patients during the rehabilitation therapeutics playing Soccer Heading (p = 0.015), Tightrope (p = 0.000), Table Tilt (p = 0.000), and Ski Slalom (p = 0.017) ( Table 4 ).

## Discussion

Multiple otoneurologic symptoms were detected in anamnesis, among them imbalance (85.7%), falls (28.5%), dizziness (17.8%), diplopia (10.7%), and tremor (7.1%). According to Teive, 1 the reported symptoms are common manifestations that may occur along the disease course. In a study conducted with people with Friedreich's ataxia, the most reported complaints of the anamnesis were uncoordinated movement (66.7%), gait imbalance (56.7%), and dizziness (50%). The cerebellum and its medial zone, while promoting the contracture of the limbic axial and proximal muscles, primarily maintain balance and stance. 18

Regarding the vestibular testing in this study, vestibular dysfunction (VD) occurred in 64.8% of participants. 35.7% featured peripheral VD and 28.6% featured central VD. This corroborates the authors' findings ^(4)^ : 50% of SCA patients were diagnosed with peripheral VD and the other 50% were diagnosed with central VD. Faryniuk et al. 19 studied 57 patients with SCA types 2, 3, 6, 7, and 10 and evidenced 72% of central VD, 12.2% of peripheral VD, and 15.8% had normal diagnosis. Nagaoka et al. 20 explained that VD combined with cerebellar atrophy significantly contributes to gait instability, an early symptom of SCA.

By comparing assessment times (T1, T2 e T3) to the VADL questionnaire domains (functional, ambulation, and instrumental), there was no significant result ( Table 1 ). Nobre et al. 21 applied the VADL questionnaire before and after ten sessions of VR in a patient with peripheral VD. The questionnaire evidenced that the patient had an initial scoring of independence in 14 activities; after rehabilitation, her results improved: there were 24 activities of independence in reassessment, thus showing a significant improvement. For Cohen, 5 the VADL questionnaire has a major importance since it aims to assess the impacts of dizziness and imbalance on the performance of ADLs. Although the current study, did not show a statistical significance, the medians decreased over time.

There was no significant result between the three assessments in the application of the ABC questionnaire, as well as in the comparison of two nominal variables between times ( Table 1 ). The ABC questionnaire assesses individuals' level of confidence to keep balance or become unstable while performing ADLs. Araújo 22 conducted a study with 14 subjects suffering from peripheral vestibulopathy and applied the ABC questionnaire before and after VR. The authors reported a statistical difference for all questionnaire items. Similar to that observed for the VADL questionnaire, the median increase over time shows a tendency for improvement after therapy. This is corroborated by the statistically significant difference observed when the scoring of the three games was assessed in the three times ( Table 4 ).

The correlation between the VADL and ABC questionnaires showed significant results for all dimensions ( Table 2 ). After the VR, patients reported improvements in body balance, which meant greater security and confidence to perform daily activities.

The correlation between ages and disease time ( Table 3 ) enabled us to observe no significant correlations between ages and the VADL and ABC questionnaires, except for the instrumental dimension of the VADL questionnaire in the T3 assessment. Regarding disease time, there was a significant result in both questionnaires, that is, the longer the disease, the worse the result. Teive 1 reported that the longer the disease, the more severe the symptoms since it is a degenerative disease.

After the 10 ^th^ virtual reality session in the current study, there was a significant improvement in the applied games, except for Ski Slalom ( Table 4 ). Hsu et al. 23 studied 70 individuals with chronic imbalance caused by Ménière disease undergoing VR sessions and observed that there was improvement in the scores of extension movements and movement coordination after the 6 ^th^ session. Negrini et al. 24 used virtual reality in a study with patients suffering from Parkinson's disease and observed that ten sessions with Nintendo Wii were enough to produce positive results in the short-term concerning the patients' balance. The VR aims at the functionality of vestibular systems by neuroplasticity mechanisms called habituation, substitution, and adaptation. This corroborates the findings of this study.

The comparison between the games played during the VR with virtual reality (T1-T3) showed significant differences in all applied games. There was an outstanding improvement for all patients undergoing rehabilitative therapeutics ( Table 4 ). Silva and Iwabe-Marchese 25 used Wii games in the VR of a child with cerebral palsy and, at the end of the research, it affected the improvement of the child's functionality. Chen et al. 26 reported a significant improvement in static and dynamic balance and in the quality of life of patients with brain lesions/cerebral vascular accidents by means of rehabilitation training with virtual reality using a Nintendo Wii ^®^ . The authors observed improvements in gaze stability after 12 sessions of exercises, 40 minutes each, for six weeks. According to the authors, the beneficial training outcomes may last for at least a month after its end.

According to the authors, 26 27 the primary functions of the vestibular system are to detect head movements, keep the image stability projected in the retinal fovea, and keep the postural control during head movements. The vestibule works by detecting head position and movement, thus providing proper sensory information to the CNS. Sensory input is primarily sent to the vestibular nuclei for processing and to the cerebellum for micro-regulation and body balance coordination. The CNS stabilizes the head and body by means of neural reflexes from the vestibular system. For the authors, 26 27 slow persecutory movements of the eye, the optokinetic function, and cervical-ocular reflexes may interact with the VOR to reduce the retinal slip.

With the recent technological breakthroughs, new sensory devices have been developed. Virtual reality therapy provides patients with a controlled environment and helps them to adapt gradually to inducing situations of dizziness, imbalance, and falls, which often affect their QOL in a significant way. 26

We would like to point out that despite failing to show statistical significance in the comparison between assessment times (T1, T2, and T3) and the applied VADL and ABC questionnaires, the median between them pointed to a tendency of improvement. Such an improvement evidenced by the application of questionnaires was not enough to show the statistical evidence. However, patients reported postural improvement, corroborated by the statistically significant differences observed when the scores of the games were assessed in the three assessment times.

Concerning the games applied, the patients evidenced a significant improvement in all assessment times.

Virtual reality is a novel rehabilitation strategy regarded as an enjoyable alternative to enhance motor recovery. Virtual reality ranges from non-immersive to fully immersive, depending on the degree to which the user is isolated from the physical surroundings when interacting with the virtual environment. The Wii® requires constant changes in standing posture from individuals, assessing their ability to control environmental stimulation using bodily changes.

The game stimulates the patient in a spontaneous and natural way to move using different motor strategies for each requested challenge in the game. Thus, the greater variability of movements in a therapeutic context and the increased attention and motivational demands are constant requirements. And that seems to provide a new and promising strategy for the rehabilitation of those patients.

Currently, it seems coherent to consider VR as a therapeutic supporting resource in the process of neurologic rehabilitation. The motivational characteristics, motion repeatability, and the induction of the directed movement to a goal permit the patient a diversity of situations and stimuli, challenging him continuously to accomplish the tasks in a therapeutic context, but there are some issues associated with such an approach that suggest caution in its use or, at the very least, further in-depth analyses due to the difficulty of equipment standardization.

In the current study, no side effects were observed, such as dizziness, sickness, or headache due to the use of the Wii® equipment. However, Park and Lee 28 assessed healthy adults and observed adverse effects while using fully immersive virtual reality with fixed and moving backgrounds. The authors reported that participants' adverse effects were reduced with fixed backgrounds. The authors 29 assessed elderly subjects suffering from Parkinson's disease, using immersive virtual reality by means of the “Oculus Rift”, and no changes were verified in the static and dynamic postural control. No discomfort was reported either.

## Conclusion

There is an effective improvement observed in the reduction of fall frequency and the improvement in balance and gait. These factors provided greater self-confidence for patients to perform ADLs, positively impacting the QOL. We emphasize the benefit of VR with virtual reality due to the symptomatology improvement in this type of population.
